# Gouty arthritis: decision-making following dual-energy CT scan in clinical practice, a retrospective analysis

**DOI:** 10.1007/s10067-018-3980-y

**Published:** 2018-01-27

**Authors:** M. Gamala, S. P. Linn-Rasker, M. Nix, B. G. F. Heggelman, J. M. van Laar, P. C. M. Pasker-de Jong, J. W. G. Jacobs, R. Klaasen

**Affiliations:** 1 Department of Rheumatology and Clinical Immunology, University Medical Center Utrecht, Box 85500, 3508 GA Utrecht, The Netherlands; 2 Department of Rheumatology, Meander Medical Center, Amersfoort, The Netherlands; 3 Department of Radiology, Meander Medical Center, Amersfoort, The Netherlands; 4 Meander Academy Meander Medical Center, Amersfoort, The Netherlands

**Keywords:** Dual-energy CT scan, Gouty arthritis, Monosodium urate

## Abstract

To establish whether dual-energy CT (DECT) is a diagnostic tool, i.e., associated with initiation or discontinuation of a urate lowering drug (ULD). Secondly, to determine whether DECT results (gout deposition y/n) can be predicted by clinical and laboratory variables. Digital medical records of 147 consecutive patients with clinical suspicion of gout were analyzed retrospectively. Clinical data including medication before and after DECT, lab results, and results from diagnostic joint aspiration and DECT were collected. The relationship between DECT results and clinical and laboratory results was evaluated by univariate regression analyses; predictors showing a *p* < 0.10 were entered in a multivariate logistic regression model with the DECT result as outcome variable. A backward stepwise technique was applied. After the DECT, 104 of these patients had a clinical diagnosis of gout based on the clinical judgment of the rheumatologist, and in 84 of these patients, the diagnosis was confirmed by demonstration of monosodium urate (MSU) crystals in synovial fluid (SF) or by positive DECT. After DECT, the current ULD was modified in 33 (22.4%) of patients; in 29 of them, ULD was started and in 1 it was intensified. Following DECT, the current ULD was stopped in three patients. In the multivariable regression model, cardiovascular disease (OR 3.07, 95% CI 1.26–7.47), disease duration (OR 1.008, 95% CI 1.001–1.016), frequency of attack (OR 1.23, 95% CI 1.07–1.42), and creatinine clearance (OR 2.03, 95% CI 0.91–1.00) were independently associated with positive DECT results. We found that the DECT result increases the confidence of the prescribers in their decision to initiation or discontinuation of urate lowering therapy regimen in of mono- or oligoarthritis. It may be a useful imaging tool for patients who cannot undergo joint aspiration because of contraindications or with difficult to aspirate joints, or those who refuse joint aspiration. We also suggest the use of DECT in cases where a definitive diagnosis cannot be made from signs, symptoms, and MSU analysis alone.

## Introduction

Gout is a disease characterized by accumulation of monosodium urate (MSU) in joints and tissues [1]. The clinical presentation varies from arthritis of one joint e.g., the first metatarsophalangeal (MTP1), to severe polyarthritis and subcutaneous tophi and sometimes tophi around tendons [2]. Gout is associated with joint damage and increased cardiovascular morbidity and mortality [3–5].

Attacks of arthritis caused by gout are very painful, and the affected persons are often not able to perform normal daily activities and work [6, 7]. Prevention with uric acid lowering drugs (ULD) of new attacks of gout and thus joint damage is an important goal of the treatment. ULD are very effective, especially if started early in the course of the disease [8–10]. Therefore, an early and accurate diagnosis of gout is crucial for targeted treatment and rapid alleviation of symptoms.

Diagnosis usually is based on clinical presentation and confirmed by demonstration of monosodium urate (MSU) crystals in synovial fluid (SF) [2, 11]. In daily clinical practice, this is usually done by blind diagnostic joint aspiration [12, 13], followed by polarized microscopy. Microscopic demonstration of MSU crystals in SF during an acute arthritis attack has sensitivity of 0.84 (95% CI, 0.77–0.92) and specificity of 0.99 to 1.00 [14, 15]. However, correct identification of crystals using polarized light microscopy in SF can be challenging [16].

Often though, the clinical presentation can be strongly suggestive of gout, whereas the aspiration is a dry tap or microscopy of the needle aspirate of SF is negative for MSU [14]. Results may be false negative due to a sampling error (no SF obtained because of incorrect placement of the needle in the affected joint, or an extra-articular location of the gout (e.g., near tendons around the joint), or incorrect microscopy, or true negative in case of a different cause of arthritis (e.g., infection, reactive arthritis).

Furthermore, aspiration may be difficult or impossible to perform in some joints.

The newest modality to image MSU deposits is dual-energy CT (DECT) scan [17–20]. The examination findings are classified as positive if urate deposition is observed on any place and as negative if no urate deposition is observed. In a systematic review [21], the pooled (95% CI) sensitivity and specificity of DECT for detecting gout were 0.87 (0.79–0.93) and 0.84 (0.75–0.90), respectively, with microscopic demonstration of MSU crystals in SF as a reference standard. DECT scanning is incorporated in the 2015 EULAR/ACR classification criteria [22]. The purpose of the current study was to analyze the clinical impact of dual-energy computed tomography (DECT) results on treatment regimen as measured by start or stop of ULD therapy after the DECT in patients with mono- or oligoarthritis possibly caused by gout. In addition, we investigated whether DECT results can be predicted by clinical, laboratory, and imaging features. Furthermore, we analyzed the false-negative DECT results, i.e., the percentage of patients with negative DECT results but a crystal proven gout diagnosis after 1 year.

## Methods

### Study design

We retrospectively evaluated medical charts of all adult patients of our outpatient clinic who underwent DECT imaging between January 2013 and December 2014 because of mono- or oligoarthritis possibly caused by gout. For patients with negative DECT result, a medical chart review was performed 1 year after DECT. The study was approved by the institutional review board of Meander Medical Center, Amersfoort, The Netherlands (15-05).

### Patients

Patient inclusion criteria were as follows: age > 18 years, DECT examination performed between January 2013 and December 2014 according to our gout protocol (see below) for clinical purposes to check the presence of uric acid crystals in or around the most affected (swollen or painful) joints.

### Study outcomes

Primary outcome is the change in ULD defined by initiation or discontinuation of one or more of the following drugs: allopurinol, benzbromarone, and febuxostat.

Secondary outcomes are as follows:

1. Prediction of DECT results by clinical, laboratory, and imaging variables
2. comparison of disease duration between patients with positive and negative DECT result
3. Percentage of false-negative DECT results defined as the clinical diagnosis crystal-proven gout after 1-year follow-up
4. Frequency of gouty attacks and uric acid levels between flares in patients with changes in therapy based on DECT

### Interventions of selected patients

#### DECT

All patients underwent DECT following the clinical suspicion of gouty arthritis by the outpatient clinic. Scans of the most affected joints and regions were made, using a dual-source DECT scanner (SOMATOM Definition Flash Dual Source CT scanner; Siemens Healthcare). The following scanning parameters were used: 140 kV and 55 mA for the one tube and 80 kV and 243 mA for the other. A two-material decomposition algorithm was performed on a multi-technique CT workspace (SW-Version VA20 Siemens Healthcare) using Syngo dual-energy Siemens Healthcare software. The material-specific difference in attenuation of urate between the two voltages allowed accurate detection of the MSU. This was color coded as green and fused with the standard greyscale CT image. DECT’s radiation dose was estimated to be 0.5 mSv per region scanned (e.g., 0.5 mSv for both hands and wrists, which are scanned together) [20]. Images were recorded as both cross-sectional and 3D images. Imaging results were classified as positive for gout if green pixilation was observed around the index joint and/or in other locations of the imaged area. A musculoskeletal radiologist, previously informed about the clinical indications for imaging, evaluated the dual-energy CT images and recorded the locations of urate deposition(s). Artifacts known to produce green pixels near a joint, i.e., nailbeds, metal prostheses, beam hardening, were excluded.

#### Testing of SF

Experienced rheumatologists (5 years or more of clinical practice) examined the synovial fluid within 1 h of sample acquisition using polarized microscopy.

### Statistical analysis

The following variables were collected: patient demographics, DECT results (positive or negative), initiation or discontinuation of ULD, frequency of gouty attacks, and uric acid levels between flares in patients with changes in therapy based on DECT. In addition, we registered clinical, laboratory, and imaging features known from the literature as predictor variables of DECT results, i.e., gender, body mass index (BMI in kg/m^2^), cardiovascular disease, diabetes mellitus, disease duration (the time in month from the start of the arthritis symptoms till the DECT), frequency of attacks (attacks per year over the past year before the DECT), uric acid levels between flares, creatinine clearance, joint involvement at the moment of DECT, MTP1 joint involvement in the past, result of microscopy (MSU crystals yes/no) around the date of the DECT, and scanned joints by DECT: hands, feet, knees, elbows, and other joints.

The 2015 EULAR/ACR classification criteria were used to score the patients (cut-off 8 points), with or without DECT [22]. In case of missing data by domain number 2 (characteristics of symptomatic episodes ever), 3 points were given.

Standard descriptive statistics were used: numerical data are given as mean ± standard deviation (SD) if normally distributed or median and interquartile range (IQR) in case of skewed distribution. DECT and microscopy results were analyzed as dichotomous data. Mann-Whitney *U* test was used to compare disease duration between patients with positive and negative DECT result. Univariable logistic regression was used to identify factors associated with positive DECT result, entering the predictors mentioned above. Odds ratios (OR) were computed with corresponding 95% confidence intervals (CI). Predictors showing a *p* < 0.10 in these univariate analyses were entered in a multiple logistic regression model with the DECT result as dependent variable. A manual backward stepwise technique was performed, removing stepwise the predictors with highest *p* value, until all *p* values were ≤ 0.1.

Mann-Whitney *U* test was used to test for statistically significant difference of disease duration between patients with positive and negative DECT result.

All statistical analyses were performed with SPSS v22.0 for Windows (SPSS, Chicago, IL, USA). A *p* value of < 0.05 is considered statistically significant.

## Results

Between 1 January 2013 and 31 December 2014, a total of 147 DECT were performed in patients with mono- or oligoarthritis possibly caused by gout. The demographic and clinical characteristics of the patients at the time of DECT are summarized in Table 1.

**Table 1:** The demographic and clinical characteristics of the patients (*n* = 147) at the time of DECT

Age, mean (SD), (years)	63.3 (13.6)
Sex, (*N*, %)	
Male	100 (68)
Female	47 (32)
Body mass index, mean (SD), (kg/m^2^)	28.5 (4.9)
Cardiovascular disease (*N*, %)	57 (39)
Diabetes mellitus (*N*, %)	21 (14.4)
Disease duration median (IQR), (years)	3 (6.6)
Frequency of attack during the past year (*N*, %)	
0–2	51 (34.5)
≥ 3	80 (54.1)
Unknown	17 (11.5)
Uric acid levels between flares, mean (SD), (μmol/l)	442.5 (124.0)
Joint involvement at the moment of DECT (*N*, %)	
MTP1	52 (62.8)
Other joints	93 (35.1)
Unknown	3 (2.0)
Result microscopy of the index joint	
Diagnostic joint aspiration of the index joint (*N*, %)	86 (58.5)
MSU crystals present (*N*, %)	25 (17.0)
MSU crystals absent (*N*, %)	61 (41.5)
Clinical evidence of tophi N, (%)	26 (17.8)
Urate lowering therapy (allopurinol, benzbromarone, and febuxostat use at the moment of DECT) (*N*, %)	
Yes	28 (19.3)
No	115 (80.0)
Unknown	4 (0.7)

Following DECT, 104 of these patients had a clinical diagnosis of gout based on the clinical judgment of the rheumatologist, and in 84 of these patients, the diagnosis was confirmed by demonstration of monosodium urate (MSU) crystals in synovial fluid (SF) or by positive DECT result. The DECT and joint aspiration results of the index joint are summarized in Table 2.

**Table 2:** DECT and joint aspiration results

	Joint fluid MSU positive (*N*, %)	Joint fluid MSU negative (*N*, %)	No joint fluid aspiration (*N*, %)	Total
Positive DECT	16 (10.88)	25 (17.0)	34 (23.12)	75 (51)
Negative DECT	9 (6.12)	36 (24.48)	27 (18.4)	72 (49)
Total	25 (17.0)	61 (41.5)	61 (41.5)	147 (100)

Eighty-six of 147 patients underwent aspiration of the index joint. Joint fluid was MSU positive in 25 patients and MSU negative in 61 patients. Twenty-five patients with synovial fluid aspirate negative for MSU had positive DECT of the index joint.

Eighty-four of 147 patients (57.14%) fulfilled the 2015 EULAR/ACR criteria for gout, 54 (36.7%) of which without taking DECT into consideration and 30 (20.4%) meeting the criteria only after a positive DECT result.

DECT scans of the most affected joints were made. Other regions were scanned too if the treating rheumatologist had requested this, e.g., based on a history of joint inflammation in this region. Table 3 shows the distribution of scanned area and the DECT results.

**Table 3:** Distribution of DECT scanned area and DECT results of 147 patients (*N*, %)

DECT scanned area	*N*, %	Positive DECT (*N*, % of all patients)
Ankles + feet	70 (47.6)	36 (24.5)
Ankles + feet + hands + wrists	28 (19.0)	13 (8.8)
Hands + wrists	17 (11.6)	5 (3.4)
Ankles + feet + hands + wrists + elbow	10 (6.8)	8 (5.4)
Ankles + feet+ knees	8 (5.4)	7 (4.8)
Knees	5 (3.4)	3 (2.0)
Elbow	3 (2.0)	0 (0)
Other (sternoclavicular, shoulders)	3 (2.0)	0 (0)
Ankles + feet + knees + hands + wrists + elbow	2 (1.4)	1 (0.7)
Hands + wrists + elbow	1 (0.7)	1 (0.7)
Total	147 (100)	74 (50.3)

### Therapeutic impact of results of DECT

The DECT result increases the confidence of the prescribers in their decision to modify urate lowering therapy regimen in 33 (22.4%) of patients. Three patients had negative DECT and no MSU crystals at joint aspiration and the urate lowering therapy was discontinued. No gouty attacks were registered in these patients after 1-year follow-up. In 29 patients, the urate lowering therapy was started, and in 1 patient, this was intensified based on the positive DECT result. One-year follow-up data were available in 21 of these patients in our outpatient clinic. In 15 of these 21 patients, the serum urate level was below 360 μmol/l (6 mg/dl), and no gouty attacks were registered in 13 of these patients.

The clinical, laboratory, and imaging variables associated with the DECT result are presented in Table 4.

**Table 4:** Univariate model analyses of factors predictive of positive DECT result

	OR (95% CI)	*p*
Gender (reference: male gender)	0.48 (0.24–0.99)	0.04
Body mass index (per kg/m^2^)	1.03 (0.96–1.11)	0.36
Cardiovascular disease yes/no	2.72 (1.36–5.42)	0.04
Diabetes mellitus yes/no	3.69 (1.26–10.71)	0.01
Urate lowering therapy use at the moment of DECT yes/no	2.6 (1.15–6.28)	0.02
Disease duration years	1.01 (1.005–1.02)	0.01
Frequency of attacks per year	1.2 (1.08–1.33)	0.01
Uric acid levels between flares (per μmol/l)	1.004 (1.001–1.007)	0.008
Creatinine clearance (per ml/min)	0.95 (0.92–0.99)	0.01
Joint involvement at the moment of DECT: MTP1 or other joints	1.69 (1.05–3.37)	0.1
Past first metatarsophalangeal (MTP1) joint involvement yes/no	3.37 (1.69–6.72)	0.01
MSU crystals at microscopy yes/no	1.62 (1.23–2.17)	0.001

Positive DECT results were significantly associated with male gender, cardiovascular disease, diabetes mellitus, ULD use at the moment of DECT, MTP1 joint involvement at the time of DECT or in the past, positive results for MSU crystals of the index joint, disease duration, frequency of attack, and uric acid levels between flares and creatinine clearance. The results of the multiple logistic regression analysis are shown in Table 5.

**Table 5:** Results of logistic regression with manual backward selection procedure

	OR (95% CI)	*p*
Cardiovascular disease yes/no	3.07 (1.26–7.47)	0.01
Disease duration (years)	1.008 (1.001–1.016)	0.03
Frequency of attack (per year)	1.23 (1.07–1.42)	0.01
Creatinine clearance (ml/min)	2.03 (0.91–1.00)	0.10

Disease duration in the DECT-positive group (median 50 months, IQR 74.7) was statistically significantly longer (*p* = 0.001) than that in the DECT-negative group (median 12 months, IQR 46).

During 1-year follow-up, 25 patients (17% of the whole group, 34.2% of the DECT negative group) with a negative DECT were diagnosed with gout based on the presence of MSU crystals in joint aspiration performed after the DECT. The mean disease duration of these patients was 2.5 years, versus 6.2 years for the remaining patient group. All patients with positive DECT results were still considered to have gout after 1-year follow-up.

## Discussion

We found that the DECT result increases the confidence of the prescribers in their decision to initiation or discontinuation of urate lowering therapy regimen in of mono- or oligoarthritis in 33 (22.4%) patients with possible gout. Thus, DECT led to earlier initiation or intensification of adequate ULD therapy 30 patients, resulting in subjective and objective relief of symptoms in 24 patients. In three patients, DECT led to avoiding unnecessary treatment. Our data suggest that for patients with uncertain diagnosis of gout, i.e., recurrent attacks of inflammatory monoarthritis or oligoarthritis but no fluid available for aspiration, negative MSU results, or joint aspiration refusal, DECT may be a useful adjunct to clinical algorithms.

To date, no study has evaluated the impact of DECT results on ULD therapy decisions in patients with suspected gouty arthritis in the outpatient clinic. Finkenstaedt et al. [23] evaluated the diagnostic impact of DECT in patients with known hyperdense soft tissue deposits on radiographs or conventional computed tomography (CT) images, so patients with high suspicion for gout. This study showed that the therapy was changed in 23/43 (53%) of the patients, with a low incidence of gouty attacks in the following year. This higher percentage compared to our study might be explained by the higher chance of gout based on prior imaging results.

In agreement with the study of Bongartz [19], we found that patients with a positive DECT had longer disease duration, which seems logical in the light of gout being a deposition disease. The diagnostic value of DECT in early gout had not yet been clearly established [19, 20]. After 1-year follow-up, 25 patients (17% of the whole group) with a negative DECT were diagnosed with gout based on the finding of MSU crystals in joint aspiration. The mean disease duration of these patients was 2.5 years compared to 6.2 years for the other patients, indicating a higher risk for false-negative DECT results in patients with shorter disease duration. This has also been found by others: in one study [19], DECT appeared to have limited sensitivity in patients with acute gout and no prior episodes of gouty arthritis.

We have to acknowledge the following study limitations: this is a retrospective study and thus diagnostic and therapeutic impact as well as follow-up data was registered through digital patient charts, with some missing data. Furthermore, there was no control group of patients who did not undergo DECT. The selection of patients undergoing DECT and the locations scanned were based on the judgment of the rheumatologist and not on well-defined criteria as the decision was made in daily clinical practice. In 61 patients, it was not possible to determine false-positive or false-negative DECT findings because of lack of the gold standard, i.e., joint aspiration. The rheumatologist tended to propose DECT more often to patients afraid of joint aspiration. In agreement with the study of Taylor [24], we reported no adverse events associated with aspiration of synovial fluid for MSU analysis. Another limitation of our study was the lack of data on the duration of ULD therapy. Finally, our study represents the experience of a single center and the diagnostic and therapeutic approach may differ in other centers. However, in our center, the therapy of patients with gout is in accordance with the current guidelines [9, 25].

In conclusion, dual-energy CT provides additional useful information to joint fluid aspiration, with impact on ULD therapy. We suggest the use of DECT in cases where a definitive diagnosis cannot be made from signs, symptoms, and MSU analysis alone. It may also be a useful diagnostic imaging modality/tool for patients who do not undergo joint aspiration because of difficult to aspirate joints, or those who refuse joint aspiration.
